# Developing a Digital Medication Adherence Intervention for and With Patients With Asthma and Low Health Literacy: Protocol for a Participatory Design Approach

**DOI:** 10.2196/35112

**Published:** 2023-04-12

**Authors:** Jasper S Faber, Charlotte C Poot, Tessa Dekkers, Natalia Romero Herrera, Niels H Chavannes, Eline Meijer, V T Visch

**Affiliations:** 1 Department of Human-Centered Design Faculty of Industrial Design Engineering Delft University of Technology Delft Netherlands; 2 Department of Public Health and Primary Care Leiden University Medical Centre Leiden Netherlands; 3 National eHealth Living Lab Leiden Netherlands; 4 Department of Psychology Health and Technology University of Twente Enschede Netherlands

**Keywords:** participatory design, low health literacy, eHealth, medication adherence, asthma, mHealth, health literacy, participatory medicine, health care, medication

## Abstract

**Background:**

Current eHealth interventions are poorly adopted by people with low health literacy (LHL) as they often fail to meet their needs, skills, and preferences. A major reason for this poor adoption is the generic, one-size-fits-all approach taken by designers of these interventions, without addressing the needs, skills, and preferences of disadvantaged groups. Participatory design approaches are effective for developing interventions that fit the needs of specific target groups; yet, very little is known about the practical implications of executing a participatory design project for and with people with LHL.

**Objective:**

This study aimed to demonstrate the application of participatory design activities specifically selected to fit the needs and skills of people with LHL and how these were manifested within an overarching eHealth design process. In addition, the study aims to present reflections and implications of these activities that could support future designers to engage people with LHL in their design processes.

**Methods:**

We used the design process of a smart asthma inhaler for people with asthma and LHL to demonstrate participatory design activities. The study was framed under 5 stages of design thinking: empathize, define, ideate, prototype, and test within 2 major iteration cycles. We integrated 3 participatory design activities deemed specifically appropriate for people with LHL: co-constructing stories, experience prototype exhibition, and video prototype evaluation.

**Results:**

Co-constructing stories was found to deepen the understanding of the participant’s motivation to use or not to use maintenance medication. This understanding informed and facilitated the subsequent development of diverse preliminary prototypes of possible interventions. Discussing these prototypes in the experience prototype exhibition helped provoke reactions, thoughts, and feelings about the interventions, and potential scenarios of use. Through the video prototype evaluation, we were able to clearly communicate the goal and functionality of the final version of our intervention and gather appropriate responses from our participants.

**Conclusions:**

This study demonstrates a participatory design approach for and with patients with asthma and LHL. We demonstrated that careful consideration and selection of activities can result in participants that are engaged and feel understood. This paper provides insight into the practical implications of participatory activities with people with LHL and supports and inspires future designers to engage with this disadvantaged target group.

## Introduction

Over the past decades, digital health (eHealth) interventions have been developed to support self-management. Such interventions can combine patient monitoring and education and include multiple behavior change strategies [1-5]. Examples of such applications are SMS text messaging systems to reinforce self-management skills, pill boxes generating alert messages when medication is missed, and interactive voice responses [6].

One specific group of people that would benefit from such interventions are people with low health literacy (LHL). A large-scale survey showed that, in Europe, nearly half of all adults reported having problems with health literacy [7]. People with LHL have problems obtaining, processing, and understanding basic health information and communicating their needs to health care professionals (HCPs) [8]. Furthermore, LHL is associated with lower patient activation. Patient activation refers to the “knowledge, skills, and confidence” of a person in managing their health and has also been called the “mindset” needed to change behavior [9-11]. This is amplified by the fact that people with LHL have differentiating illness perceptions and beliefs about their medication [12-15]. As a result, they experience difficulties in following treatment recommendations, for example taking medication as prescribed [16-18].

Approximately 50% of the people taking medication for chronic illnesses such as chronic obstructive pulmonary disease, diabetes, or cardiovascular disease are considered nonadherent [19]. Medication nonadherence has a significant impact on patients’ quality of life and has been shown to lead to poor health outcomes and increased use of health care services [20]. Medication adherence in patients with asthma is consistently low, which results in unfavorable health outcomes such as an increase in experiencing symptoms and hospitalization [21]. Previous reviews have shown promising results on the effectiveness of eHealth interventions to enhance patients’ adherence to asthma medication [6,22-24]. Yet, these interventions are mostly designed for patients with sufficient motivation, health literacy, and self-management skills and fail to address the needs, skills, and preferences of patients with LHL.

Within the design domain, it is acknowledged that involving users in the design of eHealth interventions facilitates alignment with their needs and preferences. Besides action- and community-based approaches [25], participatory design and its methods are increasingly receiving more attention. These approaches are based on the notion that when users are involved in the design and development of interventions, they are more likely to be successfully adopted [26-28]. Participatory design could uncover potential reasons for nonuse and allow designers to discover, through their participants, how technologies could be acceptable and engaging [29].

Participatory design is human centered and especially useful in the context of LHL. First, participatory methods are visual, interactive, and concrete. This benefits people who have difficulties thinking in abstract terms or who have language barriers to understand and engage with the process [26,30]. The flexibility of a participatory approach also allows to adapt and align research methods if judged inappropriate. Second, participatory methods can also bridge the gap between researchers and participants by creating a more equal and collaborative environment. This can help reduce distrust, friction, and misunderstanding that can arise due to differences in social, cultural, and economic backgrounds. Finally, a participatory design approach is iterative, which allows multiple engagements with the end user. This benefits the development of rapport and mutual trust between researcher and participant, which is known to be a strong facilitator for participant retention [31].

Nevertheless, participatory design is still seldom applied in intervention design among people with LHL. Only a few examples exist of participatory design studies on people with LHL [32,33]. The time, resource, and skill intensity of such a process, in combination with its results being difficult to generalize, decrease the attractiveness of the approach [34], and evidence regarding why and how to conduct such an approach in intervention design is falling short [35]. In addition, effectively involving disadvantaged groups, such as people with LHL, in research efforts is challenging. It has been marked by several barriers, such as participants having difficulties understanding the content of the study [36], finding it difficult to think in abstract terms [37], language or literacy problems [36], anxiety toward research or the research team [38], feelings of stigmatization [39,40], and limited exposure to technology and internet [41]. While participatory design methods have the potential to overcome these barriers, the scientific literature is unclear about which forms of participatory design can be used to develop eHealth [35]. Consequently, there is also no clear methodology on how to involve people with LHL in the participatory design process of an eHealth intervention.

Hence, the aim of this paper was to demonstrate how participatory design can be used to design an eHealth intervention that fits the needs and preferences of people with LHL. We present the development of an asthma medication adherence intervention for people with LHL to illustrate our approach.

## Methods

### Study Design

The study was conducted between February and September 2019. The study was framed under the five stages of design thinking by Hasso Plattner Institute of Design [42] and consisted of the following stages: (1) empathize to understand the user, (2) define to analyze and interpret the data, (3) ideate to explore and identify innovative solutions, (4) prototype to explore feasibility and develop a research instrument, and (5) test to evaluate usability and acceptance of the prototypes. While defined as distinct modes, in practice, the stages are iterative. This allows the researcher to reflect on previous activities and incorporate knowledge from the different stages.

**Figure 1:**
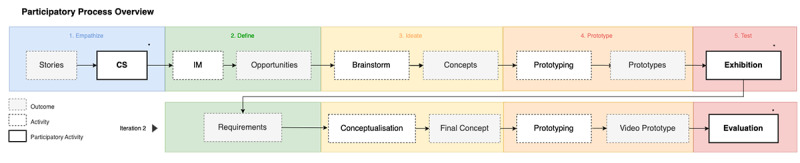
Schematic overview of the process, illustrating the different design thinking stages and their iterative character. *a participatory design activity. CS: co-constructing stories; IM: intervention mapping.

Figure 1 displays an overview of the overall design process. It shows how the 5 design thinking stages were structured across 2 major design iterations, including design activities used and outcomes generated. Throughout this paper, we distinguish between the 3 participatory design activities and the other generic design activities, with a specific focus on the former to illustrate how people with LHL can be involved in a participatory design process of an eHealth intervention. We specifically chose to embed the participatory activities at the beginning (to develop an understanding) and end (to evaluate this understanding) of the design iterations.

### Participatory Design Methods

#### Overview

Within this framework, we integrated 3 participatory activities deemed specifically effective for people with LHL. These were (1) “co-constructing stories” [43], (2) “experience prototype exhibition” [44], and (3) “video prototype evaluation” [45]. These activities were specifically selected as they would allow to effectively engage with the target group and understand their perspective.

#### Co-constructing Stories

The creation of stories helps to discover users’ thoughts and beliefs regarding a particular phenomenon. In a previous study, for example, co-constructing stories was used to gather insights regarding an interactive system to support collaboration in a meeting room [43]. Stories can be presented visually, which decreases the interview's abstractness and verbality. As such, the use of visuals has been successfully applied in other LHL-related intervention design processes as conversation starters or design tokens [46-48]. Apart from the benefits of visuals, using a fictional but relatable character in stories helps to shift the conversational focus from the individual, thereby decreasing possible anxiety-related barriers.

#### Experience Prototype Exhibition

Experience prototypes extend beyond the usability of a product and focus on understanding a person’s attitude toward a product by envisioning what it might be like to engage with it [44]. People with LHL have little prior experience regarding the use of technologies for health [41]. Using these technologies in an experience prototype evaluation session could, therefore, provoke responses and reveal attitudes toward new technological solutions that would otherwise remain undiscovered. Moreover, the physical and interactive nature of the experience prototypes allows the researcher to describe the concepts nonverbally, thereby increasing the engagement of participants with communication difficulties.

#### Video Prototype Evaluation

Paper-based prototypes are a common tool to evaluate design concepts of eHealth interventions [35]. Nevertheless, these prototypes often fail to adequately represent the concept’s core functions and interaction scenarios. A combination of paper and video prototypes would be more effective in communicating the concept toward people with LHL than paper-based prototypes alone. [45,49]. Videos have proven to be an effective tool in other intervention research and design efforts for asthma patients with LHL [50,51].

### Participants and Recruitment

The participants involved in the study included patients with asthma who have LHL and stakeholders. Patients with asthma and with LHL (n=5) were recruited by the first author and an HCP working in a disadvantaged neighborhood in The Hague, Netherlands. Qualitative and explorative approaches that aim to develop a pragmatic and in-depth understanding of a small number of participants have been argued to be effective in research approaches where not the generalizability, but the values, beliefs, and attitudes of individuals are central. This benefits the study by allowing for more flexibility and in-depth investigation of the included participants [52,53]. The patients were purposively sampled based on a self-reported diagnosis of asthma, being prescribed medication, and a subjective health literacy assessment based on the person’s characteristics (eg, migration background, occupation, educational level, and cognitive disorder) by the involved HCP. We decided to not objectively assess participants’ health literacy as this was likely to be perceived as stigmatizing and imped building a trustful relationship. The first and second authors also recruited other stakeholders, consisting of respiratory nurses (n=5), health literacy experts (n=2), design experts (n=3, TD, NRH, VTV), and eHealth researchers (n=4, NHC). These stakeholders were selected because they had long-standing experience with treating asthma, people with LHL, or participatory design methodology. We recruited 5 “language ambassadors” through an expertise center in health disparities to evaluate the final concept.

### Ethics Approval

The study protocol was cleared by the Ethical Committee of the Leiden University Medical Centre (approval number: P18.158). Informed consent was obtained prior to study participation. If written informed consent could not be given, participants provided verbal informed consent, which was recorded.

## Results

### Stage 1: Empathize

**Figure 2:**
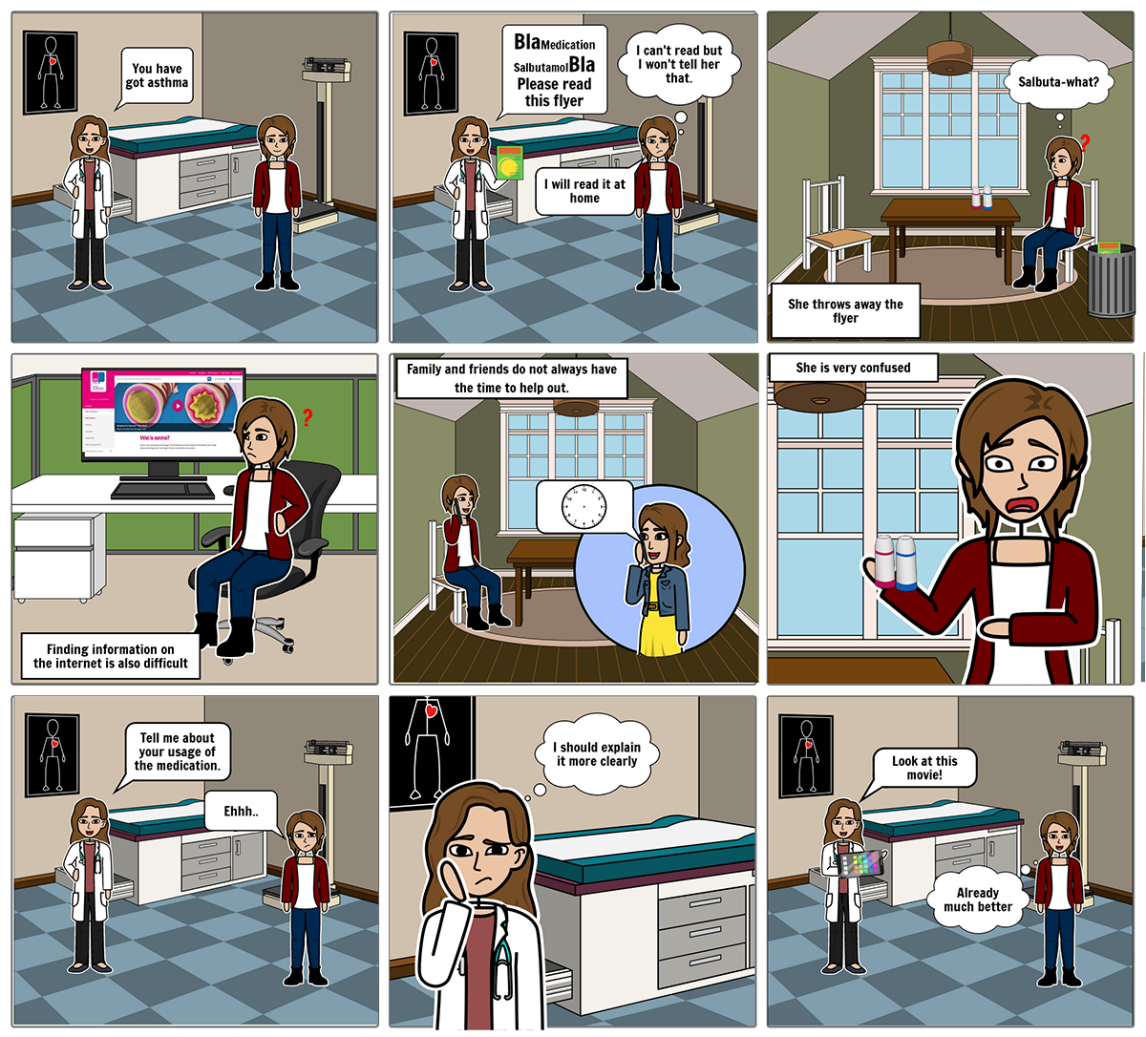
An example storyboard used during the co-constructing story sessions (translated into English).

The empathize stage served to understand the thoughts, beliefs, and perceived barriers of patients with asthma and with LHL regarding medication adherence. In this stage, we wanted to validate and discuss literature-based personas (Multimedia Appendix 1) with patients with asthma and with LHL. Personas often consist of a detailed written description [54], which was deemed suboptimal as a research tool for people with LHL as understanding and processing this type of information is often cognitively demanding for people with LHL [37]. Therefore, we converted the written persona descriptions into visual storyboards (Figure 2) using the “storyboard that tool” [55].

Two participants with asthma and LHL participated in the co-constructing stories sessions. The sessions took place at the facilities where the participants worked, lasted approximately 1 hour, and were audiorecorded. Observations and impressions about reasons for nonadherence and the co-constructed stories were collected in the form of a written report after the sessions. Using the storyboards, we asked nondirective questions such as: “How does this character experience the instructions given by the caregiver?” “How do you experience these instructions?” and “can you relate with the character and why or why not?”

The sessions helped to deepen our understanding of the preliminary insights from the initial literature review. For instance, we learned from the literature that an important reason for medication nonadherence in LHL groups is that the patients have misconceptions about the medication [14,15,56]. However, through our sessions, we gained a more nuanced view of these beliefs. The participants reported trusting their doctor’s expertise blindly, as they had difficulties understanding the purpose of the maintenance medication. Despite trusting the advice, they used their reliever inhaler instead when they experienced symptoms. When asked, participants indicated not knowing or remembering the explanations given by their HCP:

According to the doctor, I just have to use it [the medication]. That is what I know.

Male

### Stage 2: Define

We used the intervention mapping approach [57] to translate the user insights, through change objectives, toward practical design opportunities. We discussed the 22 identified change objectives (Multimedia Appendix 2) with the stakeholders and developed 3 overarching design opportunities (Table 1). In a consensus meeting with design experts, we agreed on the most feasible and important design opportunity—creating awareness about the effects of medication on symptoms through patient engagement in logging and monitoring.

**Table 1:** Design opportunities.

Design opportunity	Determinant	Description
Improve the capabilities of patients to understand and organize their medication intake behavior.	Capabilities	Empower the patient to gain authority and confidence in self-managing their asthma.
Create patient awareness about the importance and effect of the medication.	Awareness	Let the patient see the effect of the medication on the body and the relation between usage and experience of symptoms.
Change patients’ attitudes to sustain motivation over a longer period.	Attitude	Help the patient acknowledge that long-term benefits of a maintenance inhaler are as important as directly noticeable effects of the reliever inhaler.

### Stages 3 and 4: Ideation and Prototyping

The ideation and prototyping stages were directed at developing ideas and concepts that could be used to reach the design objective that resulted from the first 2 phases. To achieve this, the first author executed a brainstorming session with industrial design students to explore engagement strategies for the monitoring process (ie, monitor asthma symptoms and monitor inhaler use) and how the data can be presented to patients with LHL to promote awareness.

**Figure 3:**
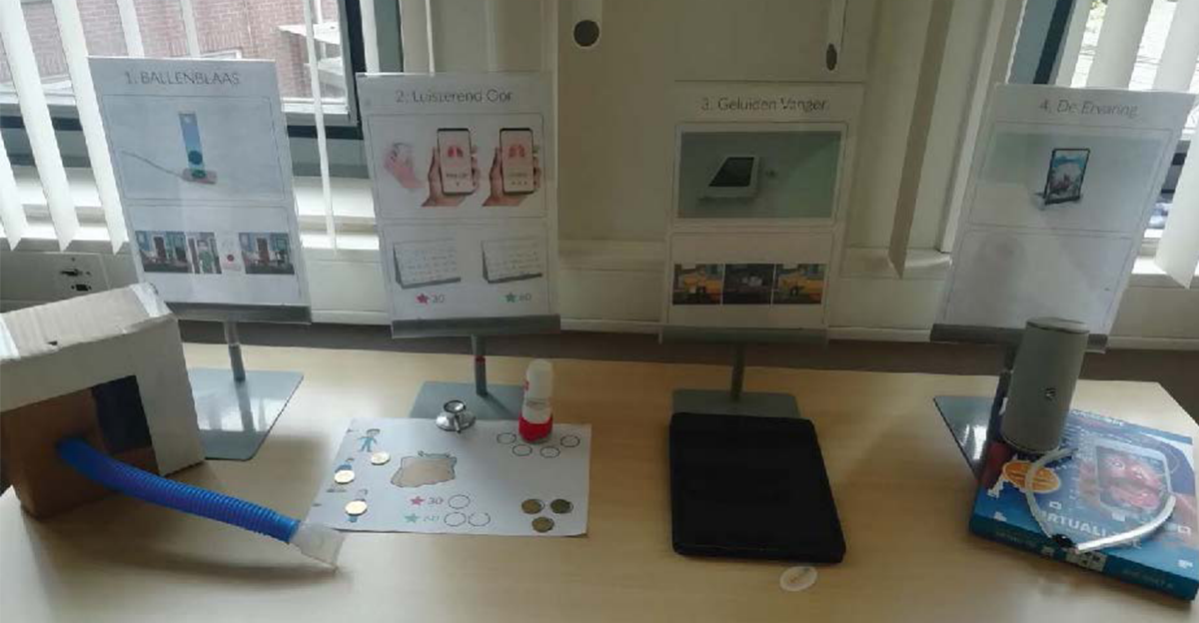
Low-fidelity prototypes and visual explainers positioned in an exhibition-style setup during the evaluation.

Four overarching design concepts resulted from these sessions, each combining multiple solution possibilities. The concepts included were (1) a smart wheeze-detecting sensor to objectively monitor asthma state, (2) an immersive experience using augmented reality to engage the user in the monitoring process, (3) a playful spirometer, and (4) a wake-up experience, displaying the result of nocturnal asthma symptoms. We translated the concepts into low-fidelity prototypes to explore their feasibility and facilitate the upcoming feedback session with the participants. The prototypes consisted of cardboard mock-ups, physical artifacts, and off-the-shelf products, such as an augmented reality T-shirt with a projection of the lungs (Figure 3).

### Stage 5: Test

Three patients with asthma and with LHL participated in the experience prototype evaluation sessions. The evaluations took place at the health facility in their neighborhood, were audiorecorded, and took approximately 1 hour. Participant responses (eg, experiences, attitudes, thoughts, and needs) for each (part of the) prototype were captured with corresponding quotations. Two days before the session, the participants received a link to a short introduction video. In the video, the researcher introduced himself and explained in lay terms the research setup. This helped set a familiar face, manage expectations, and build initial rapport. This was deemed essential to facilitate the participants’ engagement, as anxiety toward research and the research team is a common barrier in socially disadvantaged groups [36].

The session started with a brief individual interview about the demographics, living conditions, and how the participant experienced their asthma. Thereafter, the first author presented the prototypes and invited the participant to interact freely with them. Verbal and nonverbal responses were carefully observed and documented. Following initial responses, probing questions were asked, such as: “What aspects do you like about this product?” and “How do you envision yourself using this product daily?” The prototypes were discussed in random order. At the end of the session, the first author asked the participant to name the prototype or combination of prototypes they liked or did not like the most and why.

The experience prototypes were successful in provoking reactions, thoughts, and feelings about the product concepts and potential scenarios of use. Through the monitoring aspects of the concepts, we learned that participants were positive about the possibility of tracking symptoms over time, as they expected symptom tracking to give them a better understanding of their respiratory health. Through the sensor-patch included in the wake-up experience, we learned that tracking should occur almost automatically, as the participants wanted the monitoring process to be as effortless as possible.

It is just like sticking a bandage on your wound. You feel nothing, and after a while, you just remove it.

Male

Through the augmented reality experience (projecting life-like lungs on the body using augmented reality technology on a T-shirt), we learned that the participants were particularly enthusiastic about novel and innovative technologies, as they improved the perception of the product’s usefulness. The augmented reality visualization of the respiratory tract provided a realistic presentation of the lungs as “their own.” It allowed them to explore the respiratory system entirely by zooming into its various aspects, such as airways and alveoli. As one of the participants expressed:

Sometimes, I think the medication is not that important. […] Only when you really experience complaints you look for your medication. However, when you use something like this [augmented reality T-shirt], and you see it is not going well over there, you directly are going to use it. Yes, I have the feeling that now I want to use my maintenance medication.

Male, 44

Based on the gathered insights regarding the target group’s attitudes toward the prototypes, three design requirements were formulated: (1) The design should be able to objectively monitor the user’s respiratory health semiautomatically over time. (2) The design should engage the user in this monitoring process by providing a feeling that the product is innovative and useful. (3) The design should create awareness about respiratory health through feedback that is realistic and displays the respiratory system in its entirety.

### Second Iteration—The Final Concept

Following the formulated design requirements, we conducted a second iteration consisting of another ideate, prototype, and test stage to arrive at a final concept. This process consisted of concept detailing and technical design, with descriptions extending beyond this paper’s scope. The final concept aims to provide awareness through a smartphone app demonstrating data on inhaler use and asthma control. The system allows the user to zoom in on the lungs and explore relations between respiratory concepts. Simplistic icons and illustrations are used to visualize the more complicated underlying physiological processes. For example, a blue arrow that depicts a person’s asthma state is presented as the amount of air that can flow through the bronchi. Inhaler data, a proxy for underlying respiratory inflammations, are visualized as respiratory cilia being “in- or out-of-balance,” depending on the frequency of maintenance inhaler use. Hence, the maintenance medication is framed as a “helper” to bring back balance to the disturbed respiratory system.

An animation video describing the concept, its functionality, and scenarios of use was developed by the first author with Adobe Premiere Pro (Adobe) [58]. The video communicated the concept in a concise and engaging way to the participants. In addition, the first author developed paper-based visual prototypes of the key interface screens that would facilitate the discussion afterward.

For the evaluation sessions, Pharos, an expertise center familiar with developing and evaluating education material for people with LHL invited 5 people with LHL to participate in 1.5-hour interview sessions during which the prototype was discussed. A trained and experienced employee of the expertise center conducted the interviews. Each interview started with displaying the video-prototype, after which the participants were asked about their opinion and if they had any questions. Subsequently, the interface screens were presented and discussed following an interview topic guide. Interview questions included “what do you think they mean with this?” or “what do you think is presented here?” Whenever an element was unclear, we asked the participant to provide suggestions for improvement. The representative of Pharos provided a summary with recommendations for improvement after the last session. In addition, observations and participant responses by the investigator were collected in a written report.

Overall, the participants were positive about the concept as they felt that it would help them gain awareness of being nonadherent to their maintenance medication and the consequences for their lungs. The visual presentation style was understood, and the overall system was perceived as useful and innovative. However, some interface details were unclear, confusing some of the participants. For example, colors were deemed confusing when they were unrealistic (ie, a blue lung). In addition, a color-coded performance bar was suggested to visualize the improvement of the cilia.

## Discussion

### Principal Findings

This paper demonstrates a participatory design approach of a medication adherence intervention for patients with asthma and LHL. We explored the potential of applying several participatory design techniques in health intervention design for a LHL population. These consisted of co-constructing stories, an experience prototype exhibition, and a video prototype evaluation. The demonstrated activities provide novel insight in the practical use and implications of participatory design activities with people with LHL and have positive indicative value for supporting their participation in the design process.

There is a need for more insight into new and adapted methods to effectively reach and engage disadvantaged groups. Current approaches are insufficient in reaching and retaining underserved populations [36,59]. While participatory design is increasingly receiving more attention, it is still seldom applied by designers with people with LHL. Models, approaches, and guidelines for participatory design do exist; yet, they do not provide concrete steps that fit specific contexts and people. A previous study suggests there is a need for a broad range of methods that facilitate the practical application of these models [30]. The demonstration of these methods in specific contexts and target groups (ie, patients with psychiatric illness [30] and LHL) could severely improve the alignment of interventions with disadvantaged populations.

Indeed, we believe that some of the reasoning behind the activities will also apply to other disadvantaged groups. First, our activities are aimed at facilitating our participants to “tell” their stories using probes of visual scenarios and story elements [60]. Several sources on this topic state that groups experiencing communication barriers, such as people with low (health) literacy, learning difficulties, and cultural differences have difficulties understanding the purpose and contents of participatory research activities and vocalizing their thoughts and experiences [36,37]. Using scenarios and story elements as a “probe” has helped our participants in telling their stories without relying merely on verbal communication skills. In addition, the probes helped to shift the focus from the individual. This has helped our participants to become more at ease with the research setting, which could be observed based on the extensiveness of their responses. This is deemed especially helpful for groups at risk of stigmatization (ie, LHL, obesity, and mental illness) [38-40]. We propose that the nonverbal and low-threshold nature of such probes positively impacts collaboration with disadvantaged groups. Besides storyboarding and scenarios, other nonverbal participatory probing tools, such as cards, artifacts of discussion, taking pictures, and using emoticons could be equally useful [35,61,62].

Second, another facet of participatory design we applied in this project was allowing our participants to “enact” future scenarios by physically trying out new concepts [60]. Age and education are associated with having limited knowledge of and experience with health technologies [41]. Therefore, we expect that societal groups, such as people with low socio-economic status or the elderly, could experience barriers in imagining technologies and usage scenarios. “Priming” is a participatory facet that allows participants to immerse themselves in a domain [63]. Our use of experience and video prototypes has helped the participants to get a feeling of possible technologies and imagine scenarios of future use. This could be observed, for example, through the responses the augmented reality T-shirt provoked in our participants. Therefore, we propose participatory tools that facilitate interaction and immersion, such as prototypes, mock-ups, and role-play to facilitate priming for technologies.

Some aspects of the approach demonstrated in this paper could also be used in practice settings. For example, a practice nurse can use co-constructing stories to discuss multiple aspects of medication use in an easy-to-understand, nonobtrusive, and more concrete way with the patient by presenting and discussing recognizable but fictional situations. Hence, it would be interesting to explore co-constructing stories as a low-cost tool during consultations.

### Limitations

Through the participatory activities, we were able to gather important insights into the needs, skills, and preferences of people with LHL that would otherwise remain unarticulated. However, the findings of this study should be interpreted in the context of its limitations. Like most studies that address LHL, recruitment was challenging. Having practice nurses identify and invite patients for participation was effective. However, it could also have led to selection bias, resulting in, for example, people who were above average engaged with their health.

Moreover, recruitment was intensive as it required efforts to build rapport and trust and resulted in a relatively small number of participants. The small sample size should be considered regarding the representativeness of the acquired insights for the adherence intervention for patients with asthma and with LHL. In addition, researchers should be mindful in adapting the practical implications mentioned in this paper to fit their context and target group.

While the study provides insight into the practical implications of using participatory methods with people with LHL, we did not thoroughly assess the impact of this approach. Previous research has shown that participatory design can improve the process on many levels. It improves participant advocacy, trust, and sense of purpose; leads to better usability and desirability of the intervention; and achieves better health outcomes, equity, and access [64]. Therefore, future researchers could set the next step by studying if a participatory process leads to more desirable and effective health interventions for people with LHL.

An important facet of participatory design that was not integrated into our approach is allowing the participants to “make” and embody thoughts in physical artifacts [60]. In this study, the “making” stages (ie, ideating and prototyping) were done without the active involvement of people with LHL. Engaging participants in co-creating prototypes helps to generate ideas for the physical manifestation of the intervention and has been done to align interventions to the needs of disadvantaged groups [62,65]. Considering the nonverbal and tangible nature of such activities, these could have yielded fruitful interactions.

### Conclusion

In this study, we demonstrated a participatory design approach for and with people with LHL. We showed how the participatory activities could result in engagement and mutual understanding within the research process. The eHealth intervention concept resulting from this design process was perceived as an acceptable solution that creates awareness about medication adherence through understandable feedback on medication use and asthma symptoms. The participatory methods applied in this study provide a first step and inspiration for succeeding efforts to help overcome common challenges in the involvement of people with LHL in the design of eHealth interventions.

## Appendix

Multimedia Appendix 1 Fictional personality profile example.

Multimedia Appendix 2 Matrix of change.

## Abbreviations

HCP: health care professional
LHL: low health literacy

## Data Availability

The data that support the findings of this study are available from the corresponding author upon reasonable request. All data provided will be anonymized.
