# Supplementary figures and images for: Developing a Digital Medication Adherence Intervention for and With Patients With Asthma and Low Health Literacy: Protocol for a Participatory Design Approach
Source: JMIR Form Res. 2023 Apr 12;7:e35112. doi: 10.2196/35112 (PMC10134023; doi:10.2196/35112)

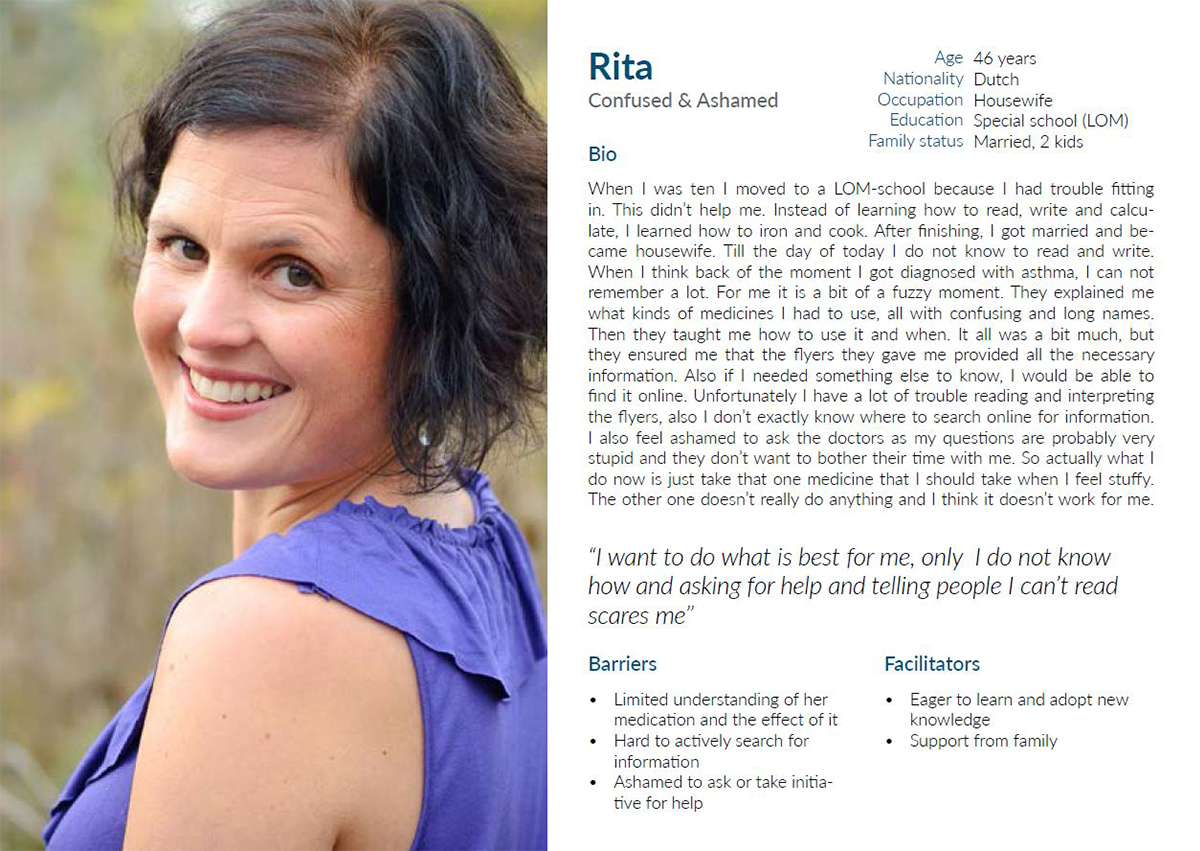

Supplement: Multimedia Appendix 1 [file formative_v7i1e35112_app1.png]

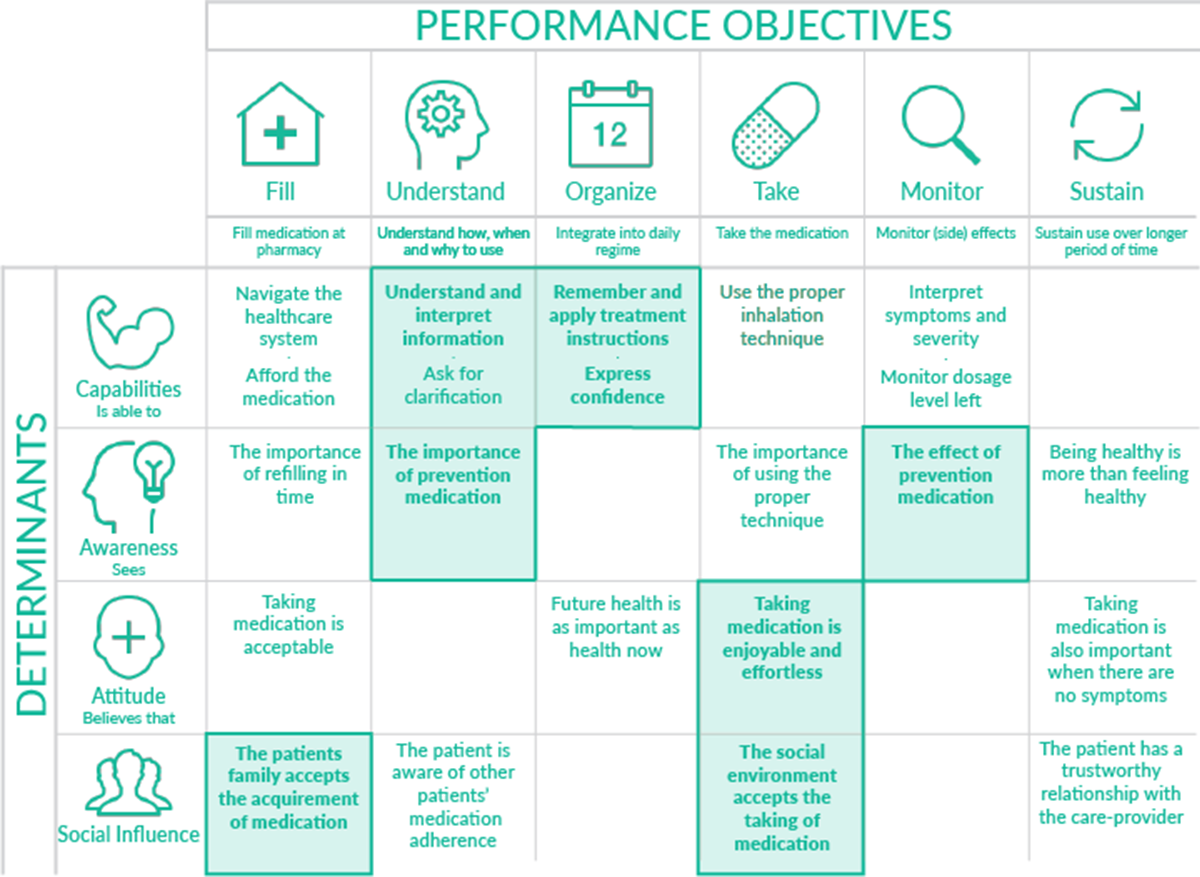

Supplement: Multimedia Appendix 2 [file formative_v7i1e35112_app2.png]
